# Real-world treatment patterns and economic burden of post-cataract macular edema

**DOI:** 10.1186/s12886-023-03113-x

**Published:** 2023-09-18

**Authors:** Gina Ahmadyar, Josh J. Carlson, Alan Kimura, Ali Alobaidi, Joelle Hallak, Ryan N. Hansen

**Affiliations:** 1 AbbVie Inc, 2525 DuPont Drive, 92612 Irvine, CA USA; 2 School of Pharmacy, University of Washington, 1956 NE Pacific St, HSB H-362, 98195 Seattle, WA USA; 3 Colorado Retina Associates, 255 S. Routt St., Suite 200, 80228 Lakewood, CO USA

**Keywords:** Post-cataract macular edema, Real world, Treatment patterns, Economic burden

## Abstract

**Background:**

Post-cataract macular edema (PCME) is a condition that can occur in patients following cataract surgery without risk factors and complications. Although 80% of patients experience spontaneous resolution after 3 to 12 months, in persistent cases, it can lead to permanent vision loss if left untreated. There are currently no standardized treatment guidelines for PCME, and there have been limited studies showing the impact of PCME on annual Medicare spending and ophthalmology-related outpatient visits per case compared to those without the complication. This study aims to evaluate real-world treatment patterns and the economic burden of patients with PCME.

**Methods:**

This retrospective claims analysis identified patients from the IBM^®^ MarketScan^®^ Commercial and Medicare Supplemental databases. Patients with (n = 2430) and without (n = 7290) PCME 1 year post cataract surgery were propensity score matched 1:3 based on age, geographic region, diabetes presence, cataract surgery type, and Charlson Comorbidity Index. Treatment pattern analysis for each PCME patient summarized the distribution of medications across lines of therapy. Economic burden analysis compared the mean number and costs of eye-related outpatient visits, optical coherence tomography imaging scans, and ophthalmic medications between the 2 groups using linear regression models.

**Results:**

Treatment pattern analysis found 27 different treatment combinations across 6 treatment lines. The most common first-line treatments were topical steroid drops (372 [30%]), topical nonsteroidal anti-inflammatory drug drops (321 [27%]), and intraocular or periocular injectable steroids (189 [15%]). Compared to match controls, PCME patients averaged 6 additional eye-related outpatient office visits (95% CI: 5.7–6.2) resulting in an additional $3,897 (95% CI: $3,475 - $4,319) in total costs. Patients filled 3 more ophthalmology-related outpatient prescription medications (95% CI: 2.8–3.2), adding $371 in total cost (95% CI: $332 – $410).

**Conclusions:**

PCME treatment patterns showed wide clinical variability in treatments and time, specifically regarding injectable treatments and combination therapy. Additionally, significantly higher healthcare resource use and economic burden were found for both patients and payers when comparing PCME patients to non-PMCE controls. These results highlight the need for treatment standardization and demonstrate that interventions targeted at preventing PCME may be valuable.

**Supplementary Information:**

The online version contains supplementary material available at 10.1186/s12886-023-03113-x.

## Background

Cataract surgery is one of the most common ocular surgeries, with an estimated 3.6 million surgeries performed each year in the United States [1]. The prevalence of cataracts is expected to rise due to the aging population, resulting in an estimated 4-fold increase in cataract surgeries [2]. Although advances in surgical techniques have improved the safety and effectiveness of these surgeries, post-cataract macular edema (PCME) can occur in the absence of complications and risk factors. PCME, also known as pseudophakic cystoid macular edema or Irvine-Gass syndrome, is believed to be caused by post-operative inflammation resulting in increased capillary permeability and fluid accumulation with subsequent cystoid changes to the retina [2, 3].

There are currently no uniform diagnosis criteria for PCME [2]. Common methods for PCME diagnosis include angiographic findings, decreased visual acuity, and optical coherence tomography (OCT). The incidence rate of uncomplicated PCME varies based on the diagnostic method and the type of cataract surgery, but is estimated to be 2.3% with modern surgical techniques [4].

PCME is self-limiting with 80% of patients experiencing spontaneous resolution after 3–12 months [5, 6]. However, persistent cases can lead to permanent vision loss if left untreated [7, 8]. PCME treatment is variable and based on provider preference because there are no standardized treatment guidelines established to date [9]. In practice, topical non-steroidal anti-inflammatory drug (NSAID) ophthalmic drops given as monotherapy or in combination with topical corticosteroids are often used first line. Studies have reported favorable outcomes with topical NSAIDs compared to topical steroids, especially NSAIDs that have enhanced penetration to the posterior segment of the eye, such as bromfenac and nepafenac [4, 8, 10]. Despite their effectiveness, some patients experience inadequate response to topical therapy and require more invasive periocular or intravitreal injectable treatments [11]. However, evidence supporting their use, and which injectable therapy is most effective, is limited.

Due to the large volume of cataract surgeries performed each year, PCME management can add up to sizable costs. One study reported that PCME increased Medicare ophthalmic payments by 85% and doubled ophthalmic charges from $5,950 to $10,410 (in 2016 US dollars) [12]. Another study reported that PCME resulted in an excess of 5.1 follow-up ophthalmologist appointments per case compared to those without the complication. These excess follow-up appointments, and the excess treatments prescribed at these appointments, resulted in a total cost of £216.81 per case ($278.03 in 2020 US dollars) [13].

There have been no studies to date assessing treatment patterns and few studies characterizing the economic consequences of PCME; therefore, this retrospective, real-world analysis of claims data aimed to evaluate the distributions of medications used for each line of therapy to depict treatment patterns for PCME and quantify the economic burden.

## Methods

### Data source

Administrative claims data was used from the IBM^®^ MarketScan^®^ Commercial and Medicare Supplemental databases. This dataset represents ~ 40 million commercially insured US adults or US adults qualifying for Medicare that have commercial supplemental insurance. The dataset is de-identified and compliant with the Health Insurance Portability and Accountability Act of 1996. The analysis was considered non-human subjects research by the Institutional Review Board (IRB) at the University of Washington and IRB approval was not required.

### Study design and patient cohorts

**Fig. 1:**
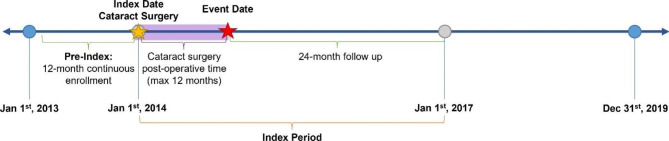
Study design and timeline. This retrospective claims analysis identified adult patients from the IBM MarketScan Commercial and Medicare Supplemental databases who had cataract surgery between 2014 and 2017

A retrospective claims analysis was performed to identify patients aged 18 years or older who had cataract surgery between 2014 and 2017. The first cataract surgery date served as each patient’s index date and was identified using Current Procedural Terminology (CPT) codes. Sample enrollment was limited to patients who had ≥ 1 year of continuous enrollment prior to their index and ≥ 3 years of continuous enrollment post index. International Classification of Diseases 9th and 10th Revision (ICD-9 and ICD-10) codes for PCME were used to separate patients into PCME and non-PCME groups. Patients in the PCME group were matched 1:3 to each non-PCME case using logistic regression to estimate propensity scores. Detailed methods for propensity score matching and a list of all procedure and diagnosis codes can be found in the Supplemental Materials (Supplemental Methods, Supplemental Tables 1–2). In the PCME group, the PCME diagnosis date served as the event date which had to occur within one year from the index date. A 1-year period was selected to ensure time for the development of PCME from cataract surgery, while accounting for flexibility in timing due to the real-world nature of the study. Time > 1 year was excluded since it is unlikely that macular edema that develops at this time point is due to cataract surgery. The number of post-operative days between the index and event date was used to determine an equivalent event date for each matched control. The full dataset contained dates between January 1, 2013, and December 31, 2019. Baseline clinical and demographic data were assessed during the year prior to or on each patient’s index date. Treatment patterns and economic burden were assessed throughout the 2-year follow-up period after each patient’s event date (Fig. 1).

### Baseline demographic and clinical characteristics

Baseline demographic characteristics and type of cataract surgery received were collected for each patient on their respective index date. Baseline clinical characteristics and comorbidities were assessed for each patient based on ICD-9 and ICD-10 codes during the pre-index period prior to cataract surgery, which also informed the calculation of a Charlson Comorbidity Index (CCI) score [14]. Outpatient pharmacy claims preceding each cataract surgery date were reviewed to identify PCME prophylactic treatments (any topical ophthalmic NSAID with or without a topical ophthalmic corticosteroid started within a week before cataract surgery). Persistent PCME was defined as the same eye or bilateral diagnosis code matched laterally at 12 months or later from the first PCME diagnosis code.

### Treatment patterns

Relevant PCME-related medications following the diagnosis event date through the 2-year follow-up period were reviewed. PCME-related treatment was defined as treatments given up to 3 months from the last outpatient service claim containing a PCME diagnosis. Included treatments consisted of medications used for PCME based on current literature (Supplemental Table 3) [9, 10]. NSAID regimens were grouped based on their absorption level to identify differences in treatment patterns [15]. Regimens containing nepafenac or bromfenac were grouped as enhanced absorption (EA) NSAIDs and others were grouped as NSAIDs. Intravitreal and sub-tenon injected steroids were grouped as injectable steroids. In-office administration of injectable treatments were identified using their respective Healthcare Common Procedure Coding System J code for active pharmacologic agent and CPT code for administration route. Each treatment line was identified as any new treatment course continued for ≥ 4 weeks. If a treatment was started within 2 weeks of a topical drop, 6 months of a steroid implant, and 3 months for all other injectable therapies, and continued together, they were considered combination therapy and grouped as the same treatment line [16, 17]. Complete changes in therapy were considered a new line of therapy for all agents regardless of timing. A subgroup analysis compared treatment patterns between PCME patients who received prophylactic therapy and those who did not.

### Economic burden

Mean counts and costs over the entire group were determined and used to provide per patient estimates. Healthcare resource utilization (HCRU) included the number of unique eye-related outpatient office visits (any visit with an eye-related provider or visits containing a PCME diagnosis code), OCT scans, and ophthalmology-related medications (number of relevant prescription medication fills and injectable treatments administered) accumulated by PCME and non-PCME patients. The number, mean, and percentage of patients who had ≥ 1 claim was recorded. Patient-related costs were those incurred by the patient and consisted of the sum of the patient co-pay, co-insurance, and deductibles. Payer-related costs were total payments made by the health plan to healthcare providers and total costs were the sum of patient and payer costs. All costs were adjusted to 2022 US dollars using the medical care component of the Consumer Price Index for all urban consumers [18]. A subgroup analysis compared economic burden between PCME patients who received prophylactic therapy and those who did not.

### Statistical analyses

Baseline characteristics were summarized for all variables using mean and standard deviation (SD) for continuous variables, and frequencies and proportions for categorical variables. Differences were assessed using Student’s *t*-tests for continuous variables, and Chi-squared or Fischer’s exact tests for categorical variables. Differences in the mean number and costs of eye-related outpatient visits, OCT scans, ophthalmic prescription medications, and injectable medication claims were assessed using multivariable linear regression models that adjusted for age, geographic region, diabetes presence, complex cataract surgery, number of cataract surgeries, and CCI score (Supplemental Methods). A sensitivity analysis using the cataract surgery date as the index date was performed for mean counts and costs (Supplemental Tables 4–5). SAS version 9.4 (SAS Institute Inc., Cary, NC, USA) was used for constructing the analytical dataset and all analyses were conducted in RStudio version 1.4.1106 (RStudio Inc., Boston, MA, USA). A 2-sided alpha with a significance level of 5% was used for all statistical comparisons.

## Results

### Study demographics

**Fig. 2:**
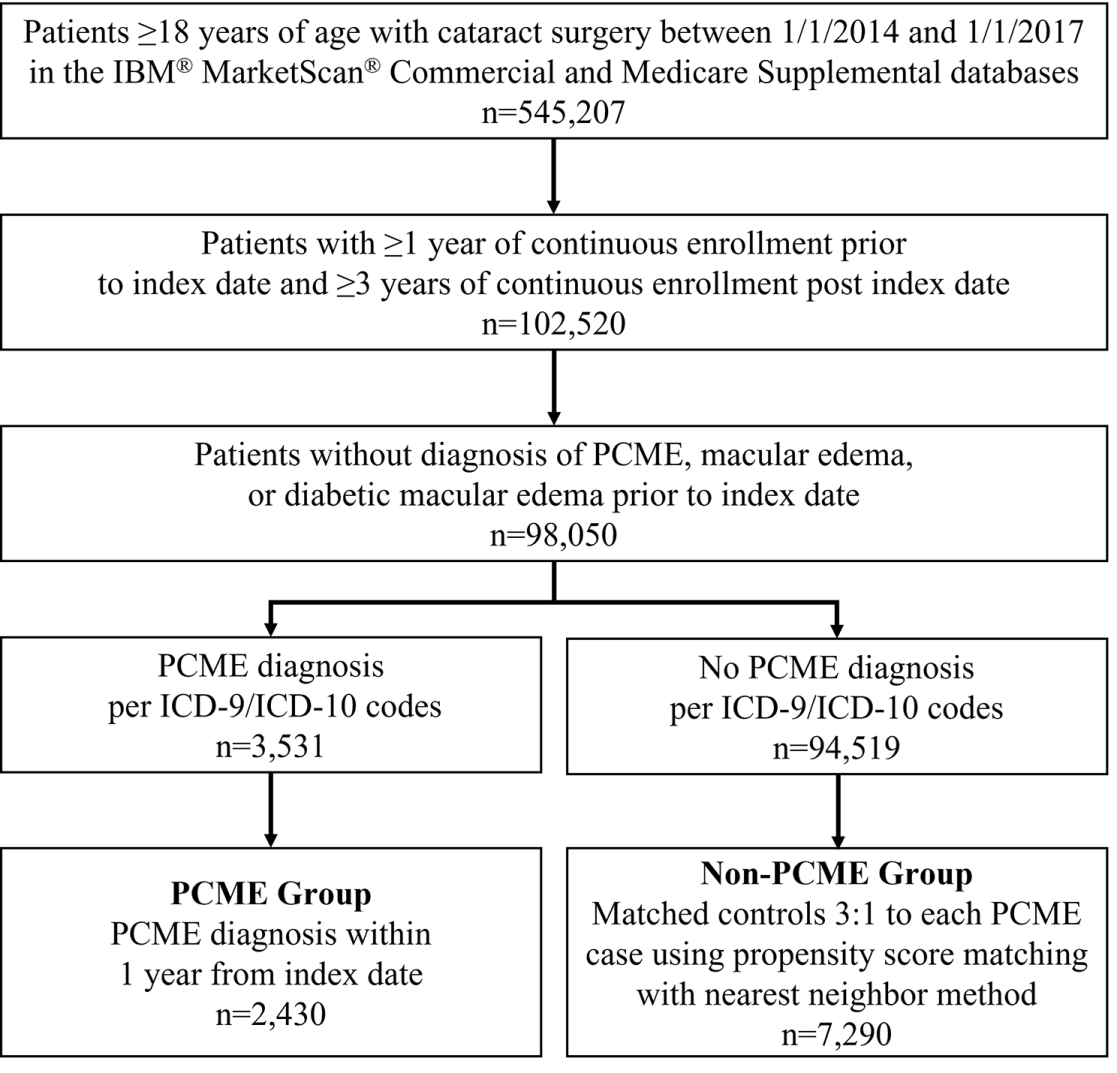
Flowchart for the enrollment of patients. ICD-9/ICD-10, International Classification of Diseases External 9th and 10th Revision; PCME, post-cataract macular edema

A total of 98,050 cataract surgery patients met the prespecified selection criteria. After assessing for a PCME diagnosis, 2,430 patients were included in the PCME group and 7,290 were matched 3:1 to form the non-PCME group (Fig. 2). A significantly higher proportion of patients in the non-PCME group had received PCME prophylaxis compared to patients in the PCME group (2,015 [27.6%] vs. 620 [25.5%], *P* = .04). Patients in the PCME group had a significantly higher prevalence of PCME risk factors (*P* < .001). There were no other statistically significant differences. The mean time from cataract surgery to PCME diagnosis was 3.6 ± 2.9 months, and 295 (12.1%) patients were found to have persistent PCME (Table 1). Baseline characteristics were examined for all individuals who were not matched (n = 88,330) and no observable differences were found.

**Table 1:** Baseline demographics and clinical characteristics

	PCME (N = 2,430)	Non-PCME (N = 7,290)	*P* value
Male sex, n (%)	1,138 (46.8)	3,294 (45.1)	*P* = .17
Age, years, mean (SD)	68 (11.4)	68 (11.3)	*P* = .94
Geographic region, n (%)			
North central Northeast South West Unknown	744 (30.6) 552 (22.7) 868 (35.7) 261 (10.7) 5 (0.3)	2,243 (30.8) 1,661 (22.8) 2,589 (35.5) 781 (10.7) 16 (0.2)	*P* = .99
Payer, n (%)			
Commercial Medicare	926 (43.1) 1,504 (56.9)	2,679 (36.7) 4,611 (63.3)	*P* = .248
Type of surgery, n (%)			
Phacoemulsification Extracapsular Intracapsular Complex	2,102 (86.5) 1 (< 0.1) 7 (0.3) 320 (13.2)	6,310 (86.6) 0 (0.1) 20 (0.3) 960 (13.2)	*P* = .49*
Prophylactic treatment, n (%)	620 (25.5)	2,015 (27.6)	***P*** **= .04**
Time from cataract surgery to PCME diagnosis, months, mean (SD)	3.6 (2.9)	N/A	N/A
Persistent PCME, n (%)	295 (12.1)	N/A	N/A
CCI Score, n (%)			
0 1 2 3+	1,408 (57.9) 456 (18.8) 309 (12.7) 257 (10.6)	4,230 (58.0) 1,371 (18.8) 917 (12.6) 772 (10.6)	*P* = .99
Mean CCI (SD)	0.87 (1.39)	0.86 (1.4)	*P* = .85
PCME risk factors, n (%)			
Diabetes Diabetic retinopathy Epiretinal membrane Retinal vein occlusion Uveitis	804 (33.1) 71 (2.9) 41 (1.7) 54 (2.2) 151 (6.2)	2,365 (32.4) 108 (1.5) 20 (0.3) 43 (0.6) 95 (1.3)	***P*** **< .001**

CCI, Charlson Comorbidity Index; PCME, post-cataract macular edema; SD, standard deviation

*Fisher’s exact test

### Treatment patterns

**Fig. 3:**
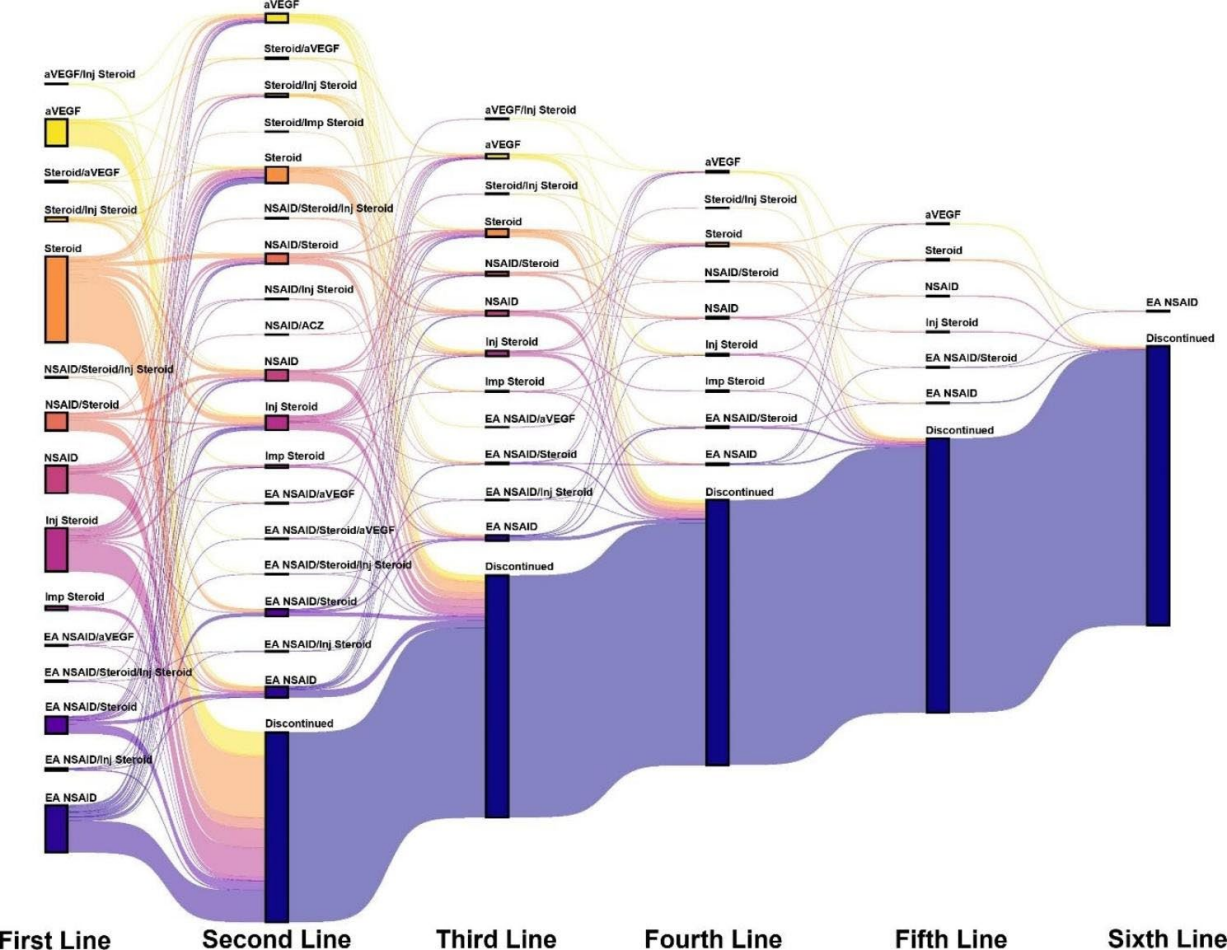
Treatment sequencing for patients in the PCME cohort across 6 lines of therapy. Each line of treatment represents regimens with > 1 count. ACZ, acetazolamide; aVEGF, anti-vascular endothelial growth factor; EA NSAID, enhanced absorption NSAID; Inj, injectable; NSAID, non-steroidal anti-inflammatory drug

Twenty-seven different combinations of medications across 6 lines of therapy were identified and added up to 1,942 treatment regimens (Supplemental Table 6). In total, 1,222 patients received ≥ 1 line of therapy. Of these individuals, 411 (34%) advanced to a second-line treatment and 47 (3.9%) received treatment beyond the fourth line. Regimens containing an EA NSAID were slightly more common than regular NSAIDs (456 vs. 373). NSAIDs were given more frequently as combination therapy with a topical steroid whereas EA NSAIDs were given more frequently as monotherapy. Otherwise, no major differences between the 2 types were observed and both were analyzed together as a class during analysis. Most patients received monotherapy (1,015 [83%]) or dual therapy (199 [16%]) first line and this trend continued across all treatment lines. The most common first-line treatments were monotherapies of topical steroids (372 [30%]), NSAIDs as a class (321 [27%]), and injectable steroids (189 [15%]). Ocular injectable containing regimens comprised 378 (31%) of first-line treatments. The most common second-line treatments included monotherapy of NSAIDs as a class (96 [24%]), dual therapy of any NSAID and topical steroid (77 [19%]), and topical steroid monotherapy (72 [18%]). Treatment sequencing across all 6 lines of therapy for any treatment line with > 1 count is shown in Fig. 3.

### Economic burden

The PCME group accumulated more HCRU (in terms of counts and costs) and had a higher proportion of patients with ≥ 1 claim across all categories compared to match controls. More patients in the PCME group had ≥ 1 eye-related outpatient provider visit compared to the control group (2,357 [97%] vs. 4,513 [62%], respectively), leading to an excess of 6 visits (95% CI: 5.7–6.2). Claims for ≥ 1 OCT scan occurred in 95% of the PCME group compared to 17% of the control group, leading to an excess of 4.5 scans (95% CI: 4.3–4.7). The PCME group received an excess of 3 prescription medications (95% CI: 2.8–3.2) and 0.9 injectable medications (95% CI: 0.7–1.0) compared to the control group. All differences were statistically significant (*P* < .0001) (Table 2).

**Table 2:** Healthcare resource use for PCME and non-PCME cohorts*

	PCME (N = 2,430)		Non-PCME (N = 7,290)		Adjusted differences (95% CI)^†^
	Patients with claim, n (%)	Mean number of claims	Patients with claim, n (%)	Mean number of claims	
Eye-related outpatient visits	2,357 (97%)	8.6	4,513 (62%)	2.7	6.0 (5.7–6.2)
Imaging (OCT)	2,310 (95%)	5.1	1,215 (17%)	0.5	4.5 (4.3–4.7)
Ophthalmology-related medications					
Prescription medications	1,591 (66%)	3.5	1,489 (20%)	0.5	3.0 (2.8–3.2)
Intraocular injectables	536 (22%)	1.1	147 (2%)	0.2	0.9 (0.7–1.0)

CI, confidence interval; OCT, optical coherence tomography; PCME, post-cataract macular edema.

*Mean number of claims were calculated over each group. Models were adjusted for age, region, diabetes presence, and CCI score. ^†^All comparisons *P* < .0001.

The PCME group incurred an additional $380 in patient out-of-pocket (OOP) costs (95% CI: $341 - $419) and $3,517 in payer costs (95% CI: $3,111 - $3,923), equaling to a mean adjusted incremental total difference of $3,897 (95% CI: $3,475 - $4,319). The difference in adjusted total costs for OCT was $295 (95% CI: $268 - $322) higher for the PCME group. Patients and payers also spent more on prescription and injectable medications which resulted in higher mean adjusted costs for both categories (prescription $371, 95% CI: $332 - $410; injectable $119, 95% CI: $99 - $140). All differences were statistically significant (*P* < .0001) (Table 3). A sensitivity analysis using the cataract surgery date as the index date for mean counts and costs was consistent with the primary analysis (Supplemental Tables 4–5).

**Table 3:** Incremental mean costs for patients and payers, and incremental mean total costs for the PCME cohort*

	PCME Incremental Mean Patient Costs (95% CI)	PCME Incremental Mean Payer Costs (95% CI)	PCME Incremental Mean Total Costs (95% CI)
Eye-related outpatient visits	$380 ($341 - $419)	$3,517 ($3,111 - $3,923)	$3,897 ($3,475 - $4,319)
Imaging (OCT)	$57 ($50 - $64)	$238 ($215 - $261)	$295 ($268 - $322)
Ophthalmology-related medications			
Prescription medications	$69 ($62 - $76)	$298 ($264 - $331)	$367 ($328 - $406)
Intraocular injectables	$9 ($6 - $12)	$111 ($90 - $131)	$119 ($99 - $140)

CI, confidence interval; OCT, optical coherence tomography; PCME, post-cataract macular edema

*Patient costs were calculated as the sum of each individual’s copay, coinsurance, and deductible. Models were adjusted for age, region, diabetes presence, and CCI score. All comparisons *P* < .0001

### Subgroup analysis

Subgroup analysis of treatment patterns of PCME patients stratified by prophylactic therapy showed that patients who received prophylactic therapy were less likely to use injectable treatments. The most common medication regimens used across all lines of therapy were topical steroid monotherapy (139 [26%]), topical NSAIDs as a class (108 [20%]), and dual therapy with both (91 [17%]). Additionally, most patients who received triple therapy or advanced to a sixth treatment line did not receive prophylactic therapy. There were no statistically significant differences in counts or costs of resources used between PCME patients who received prophylactic therapy and those who did not (Supplemental Tables 7–10).

## Discussion

This retrospective claims analysis evaluated the real-world treatment patterns and economic burden of cataract surgery patients who developed PCME compared to those who did not. PCME treatment patterns showed wide variability in the approaches to treatment and a significant economic burden for both patients and payers in the PCME group was identified.

The literature describes PCME treatment as a stepwise approach starting with topical monotherapy, then topical dual therapy, and eventually periocular or intraocular injectables as last-line options in prolonged or non-responsive cases [9, 10]. However, our analysis showed deviations from this approach. Monotherapy was observed as the most common regimen across all treatment lines and progression to topical dual therapy was not as prevalent as expected. Additionally, among first-line treatments, topical agents on their own only accounted for 56% of the medications used. The remainder comprised combination therapy and periocular or intraocular injectables, with injectable steroids being one of the most frequent treatments used [20]. The heterogeneity observed is reflective of the uncertainties surrounding PCME treatment. The consequences of this clinical variation may contribute to worse clinical outcomes and excess costs and resource use as physicians are left to trial and error with different therapies in the absence of standardized clinical guidelines. The concept of clinical variation has been identified as a component of “Failure of Care Delivery”, one of the six waste domains of the US Health Care System, which is estimated to cost in excess of $8 billion annually [19]. These findings highlight the lack of treatment consensus and a need for additional research and implementation of quality improvement initiatives for PCME treatment.

Two previous studies have explored the relationship between PCME and costs; both reported a similar directionality of outcomes to this study but were limited in sample size and follow-up time [12, 13]. This study found statistically significant differences for each HCRU category, with outpatient eye-related office visits and OCT scans having the largest differences. This excess in resource use led to significantly higher costs for both patients and payers. Eye-related outpatient visits were the main driver of additional costs incurred by PCME patients followed by outpatient prescription medications. These results demonstrate the economic burden associated with PCME. With 3.6 million cataract surgeries being performed annually in the US, which is predicted to increase with the aging population, the implications are further amplified [1, 2]. Thus, strategies focused on preventing PCME, such as prophylactic therapy, may be valuable for avoiding eye-related HCRU and could translate to cost savings on a population level.

A significantly higher proportion of patients in the control group compared to the PCME group had received prophylactic therapy and the subgroup analysis of PCME patients who used prophylactic therapy found that these patients were less likely to receive injectable treatments or advance to a sixth-line treatment. Surprisingly, comparisons of the economic burden between PCME patients who did and did not receive prophylactic therapy failed to result in any significant findings. This suggests that although prophylactic therapy may be effective at reducing the risk of PCME, once PCME develops, it may have minimal impact on reducing the long-term severity or intensity of the condition measured by incremental HCRU. Despite the latter, emphasis on prevention in clinical practice is imperative to avoid the consequences of PCME. NSAIDs have been shown to be effective in preventing PCME through their anti-inflammatory effects and ability to improve surgical efficiency by preventing pupillary miosis [4, 20]. Prophylaxis should be considered for all patients undergoing cataract surgery and especially patients with characteristics predictive of PCME development such as male sex, older age, and conditions such as epiretinal membrane, uveitis, and retinal vein occlusion [21].

A potential limitation to this study is the Marketscan^®^ database which represents individuals who have Medicare supplemental or commercial insurance. This could impact the generalizability of the results for those who are aged 65 years or older, which is the group that is more commonly affected by cataracts and most at risk for developing PCME. Additionally, because this is a claims dataset, it was not possible to confirm which eye was being treated, which may have resulted in the misclassification of treatments being grouped as combination therapy and the introduction of bias to the results. Another potential limitation was the 3 years of post-index enrollment required for inclusion and the long duration of follow-up used for the analysis. This limited the sample size and could have led to the incorporation of treatments and resources that were no longer related to PCME. The long follow-up period was chosen to account for patients who had persistent PCME and to capture any potential long-term consequences of the disease since it has been reported that patients may still experience visual consequences from PCME after resolution [20]. However, this may have resulted in an overestimation of its economic burden.

## Conclusions

Cataract surgery is one of the most frequently performed surgical procedures worldwide, making PCME a condition that can result in significant clinical and economic burden. This study found wide variability in treatment regimens used, especially with combination therapy and injectable treatments, highlighting the need for treatment standardization. These results also show that those who develop PCME following cataract surgery incur higher costs and HCRU compared to those who did not, demonstrating that interventions targeted at preventing PCME may be valuable. The need for high-quality evidence from randomized controlled trials is essential as well as the creation of best practices for the treatment of PCME to reduce variability and waste. Future studies should explore the treatment patterns and economic burden of PCME and incorporate data from sources that can provide specific details (e.g., treatment eye), such as electronic health records.

### Electronic supplementary material

Below is the link to the electronic supplementary material.

Supplementary Material 1

Supplementary Material 2

Supplementary Material 3

Supplementary Material 4

Supplementary Material 5

## Data Availability

All data generated or analyzed during this study are included in this published article and its supplementary information files.

## Abbreviations

CCI: Charslon Comorbidity Index
CPT: Current Procedural Terminology
EA: enhanced absorption
HRCU: healthcare resource utilization
ICD-9/10: International Classification of Disease 9th /10th Revision
NSAID: non-steroidal anti-inflammatory drug
OCT: optical coherence tomography
OOP: out of pocket
PCME: post-cataract macular edema
SD: standard deviation
