# Supplementary material for: Real-world treatment patterns and economic burden of post-cataract macular edema
Source: BMC Ophthalmol. 2023 Sep 18;23:380. doi: 10.1186/s12886-023-03113-x (PMC10506304; doi:10.1186/s12886-023-03113-x)
Supplement: Supplementary file 1 — Supplementary Material 1 [file 12886_2023_3113_MOESM1_ESM.docx]

**ADDITIONAL FILE 1

Supplementary Methods
*Linear Regression Modeling and Propensity Score Matching***In the PCME group, the first PCME diagnosis date served as the event date which had to occur within one year from the index date. The number of post-operative days between the index and event date was used to determine an equivalent event date for each matched control. Patients who had a diagnosis for PCME, macular edema, or diabetic macular edema prior to their index date were excluded. Logistic regression was used to calculate propensity scores to match PCME and non-PCME patients 3:1 using the nearest neighbor method, adjusting for age, geographic region, presence of diabetes, type of cataract surgery, and CCI score.

 P_i_ = p(PCME = 1| X_(1...n)_) P_j_ = p(PCME = 0 | X_(1...n)_)
 Match = min |P_i_ – P_j_|

Multivariable linear regression models that adjusted for age, geographic region, presence of diabetes, complex cataract surgery, number of cataract surgeries, and CCI score were used to calculate the differences in mean number and costs of eye-related outpatient visits, OCT scans, ophthalmic prescription medications, and injectable medication claims. Four models were used to summarize adjusted incremental differences in the counts of each resource use category. Twelve models were used to summarize adjusted incremental differences in costs of each category separately for patient, payer, and total costs.

E(Y_i_ | X_i_) = ß_0_ + ß_1_X_PCME_ + ß_2_X_age_ + ß_3_X_region_+ ß_4_X_diabetes_ + ß_5_X_complexity_ + ß_6_X_num_surg_ + ß_7_X_CCI_
