# Supplementary material for: Real-world treatment patterns and economic burden of post-cataract macular edema
Source: BMC Ophthalmol. 2023 Sep 18;23:380. doi: 10.1186/s12886-023-03113-x (PMC10506304; doi:10.1186/s12886-023-03113-x)
Supplement: Supplementary file 2 — Supplementary Material 2 [file 12886_2023_3113_MOESM2_ESM.docx]

**ADDITIONAL FILE 2

Supplementary Table 1.** ICD-9 and ICD-10 diagnosis codes.

| **ICD-9 Code/ICD-10 Code** | **Description** | **Rationale** |
| --- | --- | --- |
| H59.03, 362.53 | Cystoid macular edema | Inclusion/exclusion criteria |
| H35.35 | Cystoid macular degeneration | Inclusion/exclusion criteria |
| H35.81, 362.83 | Macular edema | Exclusion criteria |
| E11.311, E11.321, E11.331, E11.341, E11.351, E10.311, E10.321, E10.331, E10.341, E10.351, 362.07 | Macular edema associated with diabetes | Exclusion criteria |
| E11.319, E11.329, E11.339, E11.349, 362.01 | Diabetic retinopathy | Baseline characteristic |
| H35.379, 362.5 | Epiretinal membrane | Baseline characteristic |
| H34.81, 362.35 | Retinal vein occlusion | Baseline characteristic |
| H20.01, H20.02, H20.1, H43.9, H30.02, H30.03, 364.04 | Uveitis | Baseline characteristic |
| I21, I22, I25.2, 410, 412 | Acute myocardial infarction | CCI |
| I09.9, I11.0, I13.0, I13.2, I25.5, I42.0, I42.5, I42.6, I42.7, I42.8, I42.9, I43, I50, P29.0, 398.91, 402.01, 402.11, 402.91, 404.01, 404.03, 404.11, 404.13, 404.91, 404.93, 425.4-425.9, 428 | Congestive heart failure | CCI |
| I70, I71, I73.1, I73.8, I73.9, I77.1, I79.0, I79.2, K55.1, K55.8, K55.9, Z95.8, Z95.9, 093.0, 437.3, 440, 441, 443.1-443.9, 447.1, 557.1, 557.9, V43.4 | Peripheral vascular disease | CCI |
| G45, G46, H34.0, I60-I69, 362.34, 430-438 | Cerebrovascular disease | CCI |
| F00-F03, F05, G30, G31.1, 290, 294.1, 331.2 | Dementia | CCI |
| I27.8, I27.9, J68.4, J40-J47, J60-J67, J70.1, J70.3, 416.8, 416.9, 490-505, 506.4, 508.1, 508.8 | Chronic pulmonary disease | CCI |
| M05, M06, M31.5, M32, M33, M34, M35.1, M35.3, M36.0, 446.5, 710.0-710.4, 714.0-714.2, 714.8, 725 | Rheumatic disease | CCI |
| K25 – K28, 531-534 | Peptic ulcer disease | CCI |
| B18, K70.0, K70.1, K70.2, K70.3, K70.9, K71.3, K71.4, K71.5, K71.7, K73, K74, K76.0, K76.2, K76.3, K76.4, K76.8, K76.9, Z94.4, 070.22, 070.23, 070.32, 070.33, 070.44, 070.54, 070.6, 070.9, 570, 571, 573.3, 573.4, 573.8, 573.9, V42.7 | Mild liver disease | CCI |
| E10.0, E10.l, E10.6, E10.8, E10.9, E11.0, E11.1, E11.6, E11.8, E11.9, E12.0, E12.1, E12.6, E12.8, E12.9, E13.0, E13.1, E13.6, E13.8, E13.9, E14.0, E14.1, E14.6, E14.8, E14.9, 250.0-250.3, 250.8, 250.9 | Diabetes  (mild to moderate) | CCI/Baseline characteristics |
| E10.7, E11.7, E12.7, E13.7, E14.7, E10.2-E10.5, E11.2-E11.5, E12.2-E12.5, E13.2-E13.5, E14.2-E14.5, 250.4-250.7 | Diabetes with complications | CCI/Baseline characteristics |
| G04.1, G11.4, G80.1, G80.2, G81, G82, G83.0-G83.4, G83.9, 334.1, 342, 343, 344.0-344.6, 344.9 | Hemiplegia or paraplegia | CCI |
| I12.0, I13.1, N18, N19, N25.0, N03.2-N03.7, Z49, Z94.0, Z99.2, 403.01, 403.11, 403.91, 404.02, 404.03, 404.12, 404.13, 404.92, 404.93, 582, 583.0-583.7, 585, 586, 588.0, V42.0, V45.1, V56 | Renal disease | CCI |
| C43, C88, C00-C26, C30-C34, C37-C41, C45-C58, C60-C76, C81-C85, C90-C9, 140-172, 174-195.8, 200-208, 238.6 | Any malignancy, including lymphoma and leukemia, excluding malignant neoplasm of skin | CCI |
| C77, C78, C79, C80, 196-199 | Metastatic solid tumor | CCI |
| I85.0, I85.1, I85.9, I86.4, I98.2, K70.4, K71.1, K72.1, K72.9, K76.5, K76.6, K76.7, 456.0-456.2, 572.2-572.8 | Moderate to severe liver disease | CCI |
| B20, B21, B22, B24, 0.42-0.44 | HIV/AIDS | CCI |

CCI, Charlson Comorbidity Index.

**Supplementary Table 2.** Procedure codes.

| **CPT Codes** | **Description** | **Rationale** |
| --- | --- | --- |
| 66830, 66840, 66850, 66852, 66984, 66982 | Phacoemulsification | Identifying type of cataract surgery received |
| 66920, 66930,  66983 | Intracapsular | Identifying type of cataract surgery received |
| 66940 | Extracapsular | Identifying type of cataract surgery received |
| 92134 | OCT | Economic burden |

CPT, Current Procedural Terminology; OCT, optical coherence tomography.

**Supplementary Table 3.** List of treatments.

| **Topical NSAIDs** | **Carbonic anhydrase inhibitors** |
| --- | --- |
| Ketorolac tromethamine | Acetazolamide |
| Diclofenac sodium | **Injectable Steroids** |
| Nepafenac | Triamcinolone acetonide |
| Bromfenac sodium | **Implant Steroids** |
| Flurbiprofen sodium | Dexamethasone |
| **Topical Steroids** | Fluocinolone |
| Prednisolone acetate | **Injectable aVEGF** |
| Prednisolone sodium phosphate | Bevacizumab |
| Dexamethasone | Ranibizumab |
| Dexamethasone sodium phosphate | Aflibercept |
| Loteprednol etabonate | **Injectable TNF Inhibitor** |
| Difluprednate | Infliximab |
| Fluorometholone |  |
| Rimexolone |  |
| Medrysone |  |

NSAID, non-steroidal anti-inflammatory drug; TNF, tumor necrosis factor; aVEGF, anti-vascular endothelial growth factor.
