# Supplementary material for: Real-world treatment patterns and economic burden of post-cataract macular edema
Source: BMC Ophthalmol. 2023 Sep 18;23:380. doi: 10.1186/s12886-023-03113-x (PMC10506304; doi:10.1186/s12886-023-03113-x)
Supplement: Supplementary file 3 — Supplementary Material 3 [file 12886_2023_3113_MOESM3_ESM.docx]

**ADDITIONAL FILE 3

Supplementary Table 4.** Sensitivity analysis of the healthcare resource use for the PCME and non-PCME cohorts. This analysis used the date of the cataract surgery for the index date.*

|  | **PCME  (N = 2430)** | | **Non-PCME  (N = 7290)** | | Adjusted differences (95% CI) |
| --- | --- | --- | --- | --- | --- |
|  | Patients with claim (%) | Mean number  of claims | Patients with claim (%) | Mean number of claims |  |
| Eye-related outpatient visits | 2393 (98%) | 16.2 | 5794 (79%) | 7.5 | 8.7 (8.2 – 9.1) |
| Imaging (OCT) | 2349 (97%) | 6.3 | 1998 (27%) | 1.0 | 5.3 (5.1 – 5.6) |
| Ophthalmology-related medications |  |  |  |  |  |
| Prescription medications | 1792 (74%) | 3.7 | 3425 (47%) | 1.0 | 2.7 (2.5 – 2.9) |
| Intraocular injectables | 556 (23%) | 1.1 | 168 (2.3%) | 0.21 | 0.9 (0.7 – 1.0) |

CI, confidence interval; OCT, optical coherence tomography.
*Mean number of claims were calculated over each group. Models were adjusted for age, region, diabetes presence, and CCI score. All comparisons *P* < .0001.

**Supplementary Table 5.** Sensitivity analysis for incremental mean costs for patients and payors, and incremental mean total costs for the PCME cohort. This analysis used the date of the cataract surgery for the index date.*

|  | **PCME Incremental Mean Patient Costs  (95% CI)** | **PCME  Incremental Mean Payer Costs  (95% CI)** | **PCME  Incremental Mean Total Costs  (95% CI)** |
| --- | --- | --- | --- |
| Eye-related outpatient visits | $489 ($426 - $552) | $7033 ($6394 - $7671) | $7522 ($6865 - $8178) |
| Imaging (OCT) | $62 ($55 - $69) | $262 ($238 - $287) | $324 ($296 - $352) |
| Ophthalmology-related medications |  |  |  |
| Prescription medications | $42 ($37 - $47) | $183 ($164 - $202) | $225 ($202 - $247) |
| Intraocular injectables | $9 ($6 - $12) | $111 ($91 - $131) | $119 ($98 - $140) |

CI, confidence interval, OCT, optical coherence tomography.
*Patient costs were calculated as the sum of each individual’s copay, coinsurance, and deductible. Models were adjusted for age, region, diabetes presence, and CCI score. All comparisons *P* < .0001.
