# Supplementary material for: Real-world treatment patterns and economic burden of post-cataract macular edema
Source: BMC Ophthalmol. 2023 Sep 18;23:380. doi: 10.1186/s12886-023-03113-x (PMC10506304; doi:10.1186/s12886-023-03113-x)
Supplement: Supplementary file 4 — Supplementary Material 4 [file 12886_2023_3113_MOESM4_ESM.docx]

**ADDITIONAL FILE 4

Supplementary Table 6.** Treatment patterns for the PCME cohort across 6 lines of therapy.

|  | First Line  (N = 1,222) | Second Line (N = 411) | Third Line (N = 182) | Fourth Line (N = 79) | Fifth Line (N = 36) | Sixth Line (N = 11) |
| --- | --- | --- | --- | --- | --- | --- |
| Monotherapy, n (%) |  |  |  |  |  |  |
| EA NSAID | 203 (16.6) | 48 (11.7) | 24 (13.2) | 8 (10.1) | 5 (13.9) | 3 (27.3) |
| NSAID | 118 (9.7) | 48 (11.7) | 23 (12.6) | 13 (16.5) | 4 (11.1) | 1 (9.1) |
| Steroid | 372 (30.4) | 72 (17.5) | 37 (20.3) | 15 (19.0) | 7 (19.4) | 1 (9.1) |
| Inject steroid | 189 (15.5) | 65 (15.8) | 23 (12.6) | 9 (11.4) | 5 (13.9) | 1 (9.1) |
| Implant steroid | 16 (1.3) | 13 (3.2) | 6 (3.3) | 4 (5.1) | 4 (11.1) | 0 |
| aVEGF | 117 (9.6) | 42 (10.2) | 24 (13.2) | 9 (11.4) | 6 (16.7) | 1 (9.1) |
| Dual therapy, n (%) |  |  |  |  |  |  |
| EA NSAID/steroid | 73 (6.0) | 31 (7.5) | 10 (5.5) | 9 (11.4) | 2 (5.6) | 0 |
| NSAID/steroid | 77 (6.3) | 46 (11.2) | 17 (9.3) | 3 (3.8) | 1 (2.8) | 0 |
| EA NSAID/inject steroid | 10 (0.8) | 2 (0.5) | 3 (1.6) | 1 (1.3) | 0 | 1 (9.1) |
| NSAID/inject steroid | 1 (0.08) | 4 (1.0) | 1 (0.5) | 0 | 0 | 0 |
| EA NSAID/implant steroid | 0 | 0 | 1 (0.5) | 0 | 1 (2.8) | 0 |
| EA NSAID/aVEGF | 4 (0.3) | 3 (0.7) | 2 (1.1) | 1 (1.3) | 0 | 0 |
| NSAID/aVEGF | 1 (0.08) | 0 | 1 (0.5) | 0 | 0 | 0 |
| EA NSAID/ACZ | 0 | 1 (0.2) | 0 | 0 | 0 | 0 |
| NSAID/ACZ | 0 | 2 (0.5) | 0 | 0 | 0 | 0 |
| Steroid/inject steroid | 19 (1.6) | 12 (2.9) | 3 (1.6) | 3 (3.8) | 0 | 0 |
| Steroid/implant steroid | 1 (0.08) | 2 (0.5) | 0 | 1 (1.3) | 0 | 0 |
| Steroid/aVEGF | 7 (0.6) | 6 (1.5) | 1 (0.5) | 1 (1.3) | 0 | 1 (9.1) |
| Steroid/ACZ | 1 (0.08) | 1 (0.2) | 0 | 0 | 0 | 0 |
| Implant steroid/inject steroid | 0 | 1 (0.2) | 0 | 0 | 0 | 0 |
| Implant steroid/aVEGF | 0 | 1 (0.2) | 1 (0.5) | 1 (1.3) | 0 | 1 (9.1) |
| aVEGF/inject steroid | 5 (0.4) | 0 | 3 (1.6) | 1 (1.3) | 1 (2.8) | 0 |
| Triple therapy, n (%) |  |  |  |  |  |  |
| EA NSAID/steroid/inject steroid | 3 (0.2) | 3 (0.7) | 0 | 0 | 0 | 1 (9.1) |
| NSAID/steroid/inject steroid | 4 (0.3) | 4 (1.0) | 1 (0.5) | 0 | 0 | 0 |
| EA NSAID/steroid/aVEGF | 0 | 2 (0.5) | 1 (0.5) | 0 | 0 | 0 |
| NSAID/steroid/aVEGF | 1 (0.08) | 1 (0.2) | 0 | 0 | 0 | 0 |
| NSAID/steroid/ACZ | 0 | 1 (0.2) | 0 | 0 | 0 | 0 |

ACZ, acetazolamide; aVEGF, anti-vascular endothelial growth factor; EA NSAID, enhanced absorption NSAID; NSAID, non-steroidal anti-inflammatory drug.

**Supplementary Table 7.** Treatment patterns for PCME patients without prophylactic treatment across 6 lines of therapy

|  | First Line  (N = 890) | Second Line (N = 296) | Third Line (N = 131) | Fourth Line (N = 57) | Fifth Line (N = 27) | Sixth Line (N = 9) |
| --- | --- | --- | --- | --- | --- | --- |
| Monotherapy, n (%) |  |  |  |  |  |  |
| EA NSAID | 147 (16.5) | 31 (10.5) | 18 (13.7) | 7 (12.3) | 4 (14.8) | 3 (33.3) |
| NSAID | 92 (10.3) | 37 (12.5) | 15 (11.5) | 7 (12.3) | 3 (11.1) | 1 (11.1) |
| Steroid | 269 (30.2) | 54 (18.2) | 24 (18.3) | 12 (21.1) | 5 (18.5) | 1 (11.1) |
| Inject steroid | 144 (16.2) | 48 (16.2) | 17 (13.0) | 8 (14.0) | 3 (11.1) | 1 (11.1) |
| Implant steroid | 13 (1.5) | 10 (3.4) | 6 (4.6) | 4 (7.0) | 3 (11.1) | 0 |
| aVEGF | 84 (9.4) | 35 (11.8) | 18 (13.7) | 6 (10.5) | 5 (18.5) | 1 (11.1) |
| Dual therapy, n (%) |  |  |  |  |  |  |
| EA NSAID/steroid | 44 (5.0) | 21 (7.1) | 8 (6.1) | 5 (8.8) | 2 (7.4) | 0 |
| NSAID/steroid | 57 (6.4) | 29 (9.8) | 10 (7.6) | 1 (1.8) | 1 (3.7) | 0 |
| EA NSAID/inject steroid | 8 (0.9) | 2 (0.7) | 2 (1.5) | 1 (1.8) | 0 | 1 (11.1) |
| NSAID/inject steroid | 1 (0.1) | 3 (1.0) | 1 (0.8) | 0 | 0 | 0 |
| EA NSAID/implant steroid | 0 | 0 | 1 (0.8) | 0 | 1 (3.7) | 0 |
| EA NSAID/aVEGF | 2 (0.2) | 1 (0.3) | 2 (1.5) | 1 (1.8) | 0 | 0 |
| NSAID/aVEGF | 0 | 0 | 1 (0.8) | 0 | 0 | 0 |
| EA NSAID/ACZ | 0 | 1 (0.3) | 0 | 0 | 0 | 0 |
| NSAID/ACZ | 0 | 2 (0.7) | 0 | 0 | 0 | 0 |
| Steroid/inject steroid | 14 (1.6) | 7 (2.4) | 3 (2.3) | 3 (5.3) | 0 | 0 |
| Steroid/implant steroid | 1 (0.1) | 1 (0.3) | 0 | 1 (1.8) | 0 | 0 |
| Steroid/aVEGF | 3 (0.3) | 4 (1.4) | 1 (0.8) | 0 | 0 | 1 (11.1) |
| Steroid/ACZ | 1 (0.1) | 1 (0.3) | 0 | 0 | 0 | 0 |
| Implant steroid/inject steroid | 0 | 1 (0.3) | 0 | 0 | 0 | 0 |
| Implant steroid/aVEGF | 0 | 0 | 1 (0.8) | 1 (1.8) | 0 | 0 |
| aVEGF/inject steroid | 4 (0.4) | 0 | 3 (2.3) | 0 | 0 | 0 |
| Triple therapy, n (%) |  |  |  |  |  |  |
| EA NSAID/steroid/inject steroid | 3 (0.3) | 3 (1.0) | 0 | 0 | 0 | 0 |
| NSAID/steroid/inject steroid | 2 (0.2) | 3 (1.0) | 0 | 0 | 0 | 0 |
| EA NSAID/steroid/aVEGF | 0 | 1 (0.3) | 0 | 0 | 0 | 0 |
| NSAID/steroid/aVEGF | 1 (0.1) | 0 | 0 | 0 | 0 | 0 |
| NSAID/steroid/ACZ | 0 | 1 (0.3) | 0 | 0 | 0 | 0 |

ACZ, acetazolamide; aVEGF, anti-vascular endothelial growth factor; EA NSAID, enhanced absorption NSAID; NSAID, non-steroidal anti-inflammatory drug.

**Supplementary Table 8.** Treatment patterns for PCME patients with prophylactic treatment across 6 lines of therapy

|  | First Line (N = 332) | Second Line (N = 115) | Third Line (N = 51) | Fourth Line (N = 22) | Fifth Line (N = 9) | Sixth Line (N = 2) |
| --- | --- | --- | --- | --- | --- | --- |
| Monotherapy, n (%) |  |  |  |  |  |  |
| EA NSAID | 56 (16.9) | 17 (14.8) | 6 (11.8) | 1 (4.5) | 1 (11.1) | 0 |
| NSAID | 26 (7.8) | 11 (9.6) | 8 (15.7) | 6 (27.3) | 1 (11.1) | 0 |
| Steroid | 103 (31.0) | 18 (15.7) | 13 (25.5) | 3 (13.6) | 2 (22.2) | 0 |
| Inject steroid | 45 (13.6) | 17 (14.8) | 6 (11.7) | 1 (4.5) | 2 (22.2) | 0 |
| Implant steroid | 3 (0.9) | 3 (2.6) | 0 | 0 | 1 (11.1) | 0 |
| aVEGF | 33 (9.9) | 7 (6.1) | 6 (11.8) | 3 (13.6) | 1 (11.1) | 0 |
| Dual therapy, n (%) |  |  |  |  |  |  |
| EA NSAID/steroid | 29 (8.7) | 10 (8.7) | 2 (3.9) | 4 (18.2) | 0 | 0 |
| NSAID/steroid | 20 (6.0) | 17 (14.8) | 7 (13.7) | 2 (9.1) | 0 | 0 |
| EA NSAID/inject steroid | 2 (0.6) | 0 | 1 (2.0) | 0 | 0 | 0 |
| NSAID/inject steroid | 0 | 1 (0.9) | 0 | 0 | 0 | 0 |
| EA NSAID/aVEGF | 2 (0.6) | 2 (1.7) | 0 | 0 | 0 | 0 |
| NSAID/aVEGF | 1 (0.3) | 0 | 0 | 0 | 0 | 0 |
| Steroid/inject steroid | 5 (1.5) | 5 (4.3) | 0 | 0 | 0 | 0 |
| Steroid/implant steroid | 0 | 1 (0.9) | 0 | 0 | 0 | 0 |
| Steroid/aVEGF | 4 (1.2) | 2 (1.7) | 0 | 1 (4.5) | 0 | 0 |
| Implant steroid/aVEGF | 0 | 1 (0.9) | 0 | 0 | 0 | 1 (50.0) |
| aVEGF/inject steroid | 1 (0.3) | 0 | 0 | 1 (4.5) | 1 (11.1) | 0 |
| Triple therapy, n (%) |  |  |  |  |  |  |
| EA NSAID/steroid/inject steroid | 0 | 0 | 0 | 0 | 0 | 1 (50.0) |
| NSAID/steroid/inject steroid | 2 (0.6) | 1 (0.9) | 1 (2.0) | 0 | 0 | 0 |
| EA NSAID/steroid/aVEGF | 0 | 1 (0.9) | 1 (2.0) | 0 | 0 | 0 |
| NSAID/steroid/aVEGF | 0 | 1 (0.9) | 0 | 0 | 0 | 0 |

aVEGF, anti-vascular endothelial growth factor; EA NSAID, enhanced absorption NSAID; NSAID, non-steroidal anti-inflammatory drug.
