# Supplementary material for: Real-world treatment patterns and economic burden of post-cataract macular edema
Source: BMC Ophthalmol. 2023 Sep 18;23:380. doi: 10.1186/s12886-023-03113-x (PMC10506304; doi:10.1186/s12886-023-03113-x)
Supplement: Supplementary file 5 — Supplementary Material 5 [file 12886_2023_3113_MOESM5_ESM.docx]

**ADDITIONAL FILE 5

Supplementary Table 9.** Healthcare resource use for the PCME cohort stratified by prophylaxis use*

|  | **Prophylaxis  (N = 620)** | | **No Prophylaxis  (N = 1810)** | | Adjusted differences (95% CI) |
| --- | --- | --- | --- | --- | --- |
|  | Patients  with claim  n (%) | Mean number  of claims | Patients  with claim  n (%) | Mean number of claims |  |
| Eye-related outpatient visits | 603 (97%) | 8.4 | 1754 (97%) | 8.7 | -0.4 (-1.0 - 0.3) |
| Imaging (OCT) | 586 (95%) | 5.0 | 1724 (95%) | 5.1 | 0.0 (-0.4 - 0.4) |
| Ophthalmology-related medications |  |  |  |  |  |
| Prescription medications | 431 (70%) | 3.9 | 1160 (64%) | 3.3 | 0.5 (-0.1 - 1.0) |
| Intraocular injectables | 146 (25%) | 1.1 | 390 (22%) | 1.0 | 0.1 (-0.2 - 0.4) |

CI, confidence interval; OCT, optical coherence tomography.
*Mean number of claims were calculated over each group. Models were adjusted for age, region, diabetes presence, type of cataract surgery, number of cataract surgeries, and CCI score. All comparisons *P* > .05.

**Supplementary Table 10.** Mean patient, payer, and total costs for the PCME cohort who received prophylactic therapy*

|  | **Prophylaxis Incremental Mean Patient Costs  (95% CI)** | **Prophylaxis Incremental Mean Payer Costs  (95% CI)** | **PCME Incremental Mean Total Costs  (95% CI)** |
| --- | --- | --- | --- |
| Eye-related outpatient visits | $65 (-$36 - $168) | $221 (-$632 - $1074) | $287 (-$604 - $1178) |
| Imaging (OCT) | -$11 (-$25 - $2) | -$13 (-$66- $41) | -$24 (-$83 - $35) |
| Ophthalmology-related medications |  |  |  |
| Prescription medications | $7 (-$9 - $24) | $95 (-$4 - $193) | $102 (-$9 - $213) |
| Intraocular injectables | $10 (-$2 - $21) | $31 (-$13 - $75) | $41 (-$7 - $88) |

CI, confidence interval, OCT, optical coherence tomography.
*Patient costs were calculated as the sum of each individual’s copay, coinsurance, and deductible. Models were adjusted for age, region, diabetes presence, type of cataract surgery, number of cataract surgeries, and CCI score. All comparisons *P* > .05.
